# Weather Conditions Are Systematically Associated With Long‐Range Nonroutine Movements in a Large Scavenger

**DOI:** 10.1002/ece3.73434

**Published:** 2026-04-09

**Authors:** Jacopo Cerri, Ilaria Fozzi, Davide De Rosa, Chiara Costantino, Mauro Aresu, Dionigi Secci, Marco Muzzeddu, Fiammetta Berlinguer

**Affiliations:** ^1^ Mammal Research Institute, Polish Academy of Sciences Bialowieza Poland; ^2^ Dipartimento di Medicina Veterinaria Università degli Studi di Sassari Sassari Italy; ^3^ Via Crispi 5 Macomer Italy; ^4^ Agenzia Forestale Regionale per lo Sviluppo del Territorio e l'Ambiente della Sardegna (Fo.Re.S.T.A.S.) Cagliari Italy

**Keywords:** biologging, foraging, forays, scavengers, soaring birds, vultures

## Abstract

Movement data are valuable for the conservation of large soaring birds, as these move across large distances and experience a wide range of threats. As species like Old World vultures rely on soaring flight, weather conditions are crucial for their movements. However, no study explored the extent to which weather conditions can predict long‐range nonroutine movements, such as those associated with forays, prospecting, or dispersal. We fitted Generalized Additive Mixed Models to predict the probability that Griffon Vultures ( *Gyps fulvus* , n. individuals = 20, n. GPS locations = 168,202) living in Sardinia (Italy) engaged in short‐range, as well as in medium‐ and long‐range movements outside their home range, under different weather conditions, in terms of solar radiation, wind direction, and wind strength. Griffon Vultures restricted their movements around the colony under very weak winds and were less prone to venture outside their home range under very strong winds. Medium and long‐range movements outside the home range were more common for northwestern and southeastern winds of intermediate strength, in conditions of good solar radiation. However, the duration of long‐range movements increased with decreasing solar radiation. This might indicate that wind sometimes displaces Griffon Vultures and scarce solar radiation then prevents them from returning to the colonies, forcing them to engage in long journeys across unfamiliar landscapes. Our findings indicate that some types of long‐range nonroutine movements in vultures are not entirely intentional, but rather triggered by weather conditions. Combining high‐resolution movement and weather data could allow researchers to predict these movements in advance and adaptively increase data acquisition from GPS tags to study vulture behavior during nonroutine movements and improve conservation actions.

## Graphical abstract

As vultures rely on soaring flight, the interplay of solar radiation, as well as wind direction and strength, is crucial for both short‐ and long‐range movements. Under very weak wind conditions, Griffon Vultures restricted their movements in the areas around the colony, as exploring areas at the borders of their home range is more demanding. Conversely, under very strong winds, extra‐home range movements were uncommon, as Griffon Vultures could be less prone to venture outside well‐known areas. Our findings indicate that some types of nonroutine movements in vultures are not entirely intentional, and weather conditions can play a crucial role in triggering them. Combining high‐resolution movement and weather data could allow researchers to study them in advance and adaptively improve data acquisition from GPS tags to study vulture behavior during nonroutine movements and improve conservation actions.

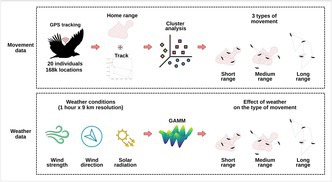

## 1 Introduction

Movement data are increasingly used to attain a sounder understanding of animal ecology (Nathan et al. 2022) and inform conservation policies (Allen and Singh 2016). Information derived from movement data is particularly valuable for the conservation of taxa such as Old World vultures (Ogada et al. 2012), which can move across large distances and therefore experience a wide range of threats.

Satellite telemetry (Duriez et al. 2014) shed light on the bioenergetics of flight in vultures (Duriez et al. 2014), as well as on the role played by social and heterospecific cues (Oliva‐Vidal et al. 2024; Sassi et al. 2024). For several species of vultures, this technology also allowed to study long‐range movements that occur periodically throughout the life of an individual, like seasonal migrations, (Arkumarev et al. 2019; Buechley et al. 2018; Kang et al. 2019; Martínez et al. 2024), as well as long‐range movements occurring irregularly (hereinafter referred as “nonroutine” movements; like explorative dispersal, García‐Macía et al. 2023; Martínez et al. 2025; Tréhin et al. 2024; Tobajas et al. 2024). This knowledge, in turn, revealed the behavioral consequences of captive breeding (Jobson et al. 2021; Margalida et al. 2013) and release methods (Cerri, De Rosa, Aresu, et al. 2024; Fozzi et al. 2023; Rousteau et al. 2022), predicted exposure to potential threats (Cervantes et al. 2023; Morant et al. 2024) and informed the design of protection areas (Kane et al. 2022) and ecotourism (Fozzi et al. 2025).

A frontier in research about the movement ecology of vultures is the integration between high‐resolution environmental data and movement data, to assess the role played by weather conditions on the motion capacity of these species (Nathan et al. 2008). Soaring birds like vultures are strongly dependent on uplifts, which arise from the interplay of air temperature, terrain morphology, and wind conditions (Scacco et al. 2019, 2023). However, the few existing studies exploring the link between weather conditions and vulture movements focused on the influence of solar radiation (Rivers et al. 2014; Poessel, Brandt, Mendenhall, et al. 2018), with significant gaps still surrounding the role played by wind, such as the interplay between wind direction and strength. Addressing this gap would be particularly valuable from an ecological perspective.

Given the high energetic cost of flapping flight in vultures (Duriez et al. 2014), short‐range movements, such as foraging around colonies, should be performed under optimal wind conditions (Alerstam et al. 2019). However, no study tested the extent to which vultures can engage in short‐range movements under optimal conditions in terms of wind direction but suboptimal wind strength, and vice versa.

Moreover, for long‐range movements, the effect of wind conditions has been assessed only for migratory movements (Efrat et al. 2023; Vidal‐Mateo et al. 2016), ignoring nonroutine movements (e.g., forays, Conradt et al. 2003; e.g., prospecting, Chaubet et al. 2025). Even the informed dispersal theory, perhaps the most influential framework for this type of movement (Reed et al. 1999), ignores the role of wind conditions entirely. However, empirical evidence indicates that at least on one occasion strong winds promoted the dispersal and colonization of an island by Griffon Vultures (Tavecchia and Cortés‐Avizanda 2021) and that optimal winds facilitate prospecting in other large soaring raptors (e.g., *Aquila chrysaetos* , Poessel et al. 2022). There is therefore a need to address the systematic effect of wind conditions, and their interplay with radiation, as a proximate driver of long‐range nonroutine movements in vultures.

In this study, we explored how different wind conditions affected the probability that Griffon Vultures ( *Gyps fulvus* ) engaged in short‐range and long‐range movements in a non‐migratory population. Our findings indicate that weather conditions are consistently associated with different types of movements in Griffon Vultures, and it is reasonable to hypothesize that some long‐range movements occur because individuals are first displaced by strong winds and then face unsuitable weather conditions, preventing them from returning to the colony.

## 2 Methods

### 2.1 Study Area

**FIGURE 1:**
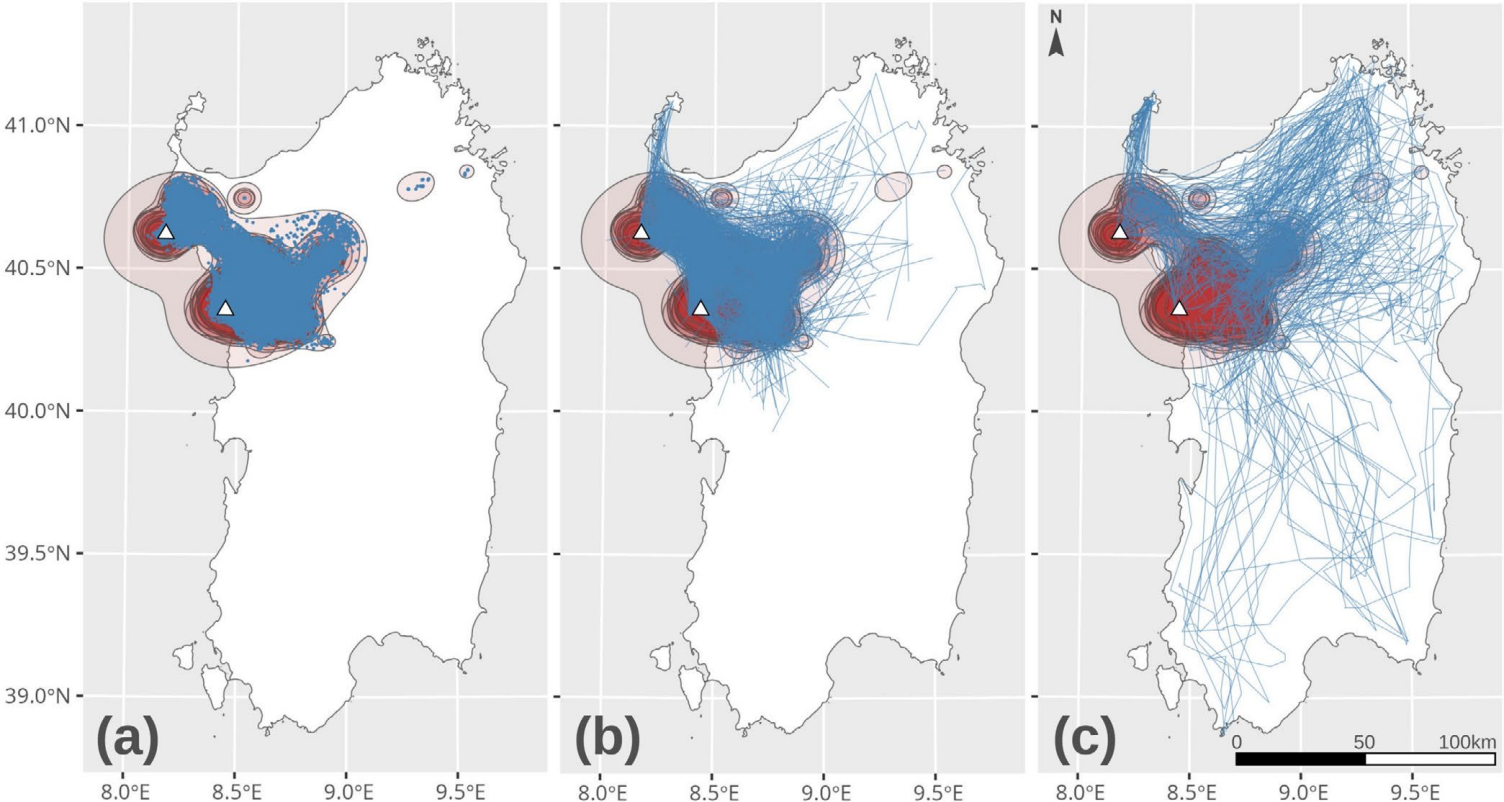
Map of the study area with 95% isopleths of individual home ranges (HR, in red) and the three types of movement obtained from cluster analysis: Short‐range movements (left), medium‐range movements (center), and long‐range movements (right). The two colonies are represented as triangles.

The study area includes the westernmost part of Sardinia (Italy), the second largest island of the Mediterranean Sea (Figure 1). Due to its Mediterranean climate, rainfalls are scarce and concentrated between November and December (Fratianni and Acquaotta 2017). The most common winds are the Mistral, which comes from the northwest, and the Sirocco, which comes from the southeast (Furberg et al. 2002).

Sardinia hosts a population of 424–470 (86 breeding pairs) Griffon Vultures (Berlinguer et al. 2024). Griffon Vultures roost, nest, and forage around two main colonies in the northwest of the island, near the municipalities of Bosa and in Porto Conte Regional Park (Figure 1, Cerri et al. 2023). Griffon Vultures have smaller home ranges than their conspecifics in mainland Europe (Cerri et al. 2023). Contrary to Griffon Vultures from other areas of Europe, which migrate to sub‐Saharan Africa in their first year of age (Arkumarev et al. 2019; Martínez et al. 2024) or which move seasonally in response to transhumance (Aguilera‐Alcalá et al. 2022), long‐range routine movements have never been observed in individuals from the Sardinian population, similarly to Griffon Vultures living in other Mediterranean islands, such as Crete or Mallorca (Xirouchakis and Mylonas 2004).

### 2.2 Data Collection and Preparation

Between 2016 and 2022, 79 Griffon Vultures were released in a conservation translocation program within the Life Under Griffon Wings project (LIFE14 NAT/IT/000484), to enhance the viability of the Sardinian population (Aresu et al. 2022). In line with requirements about translocation programs for wildlife species, the manipulation, marking, and release of Griffon Vultures were authorized by competent authorities. These included the province of Sassari (permit n. 3079, issued on the 08/03/2016), the environmental secretariat of Regione Sardegna (permit n. 6198, issued on the 29/03/2016), the Italian Ministry for the Environment (permit n. 14500 issue on the 07/07/2016), the Italian Ministry for Agriculture and Forestry (permit n. 14655, issued on the 13/06/2016) and the National Institute for Environmental Protection and Research (ISPRA, permit n. 13368TC2 issued on the 23/02/2016).

Released individuals had been recovered from the wild in the Iberian Peninsula, or had been bred under different captivity breeding programs (LIFE14 NAT/IT/000484). Before being released in the wild, Griffon Vultures were tested for pathogens and lead poisoning and then kept at acclimatization aviaries so that they could familiarize themselves with the release site (Fozzi et al. 2023). They were also fitted with engraved metal rings from Istituto Superiore per la Protezione e la Ricerca Ambientale (ISPRA), placed on one tarsus, and with a plastic colored ring on the other tarsus to facilitate their identification. Necessary permits for handling and tagging vultures were obtained from the Sardinian Regional Authority and ISPRA. The transmitters were fitted following the best practice in animal welfare—the heads of the birds were covered to guarantee minimal stress, and the handling time was reduced to less than 10 min. Before being released, 45 individuals were also equipped with solar‐powered GPS/GSM transmitters, attached with a Teflon leg‐loop harness comprised of 3 assembled strings (round silicone cord 2 mm + tubular Teflon ribbon 0.25 and 0.44), following Hegglin and Aebischer (2004). GPS tags were programmed to collect the location of released Griffon Vultures from dawn (approx. 6 a.m.) to dusk (approx. 6 p.m.), with a frequency of data acquisition that depended upon solar power (Figure S1). In this study, we used locations from 20 individuals (n. GPS fix = 168,202), selected based on a minimum tracking duration of 300 days to ensure full seasonal coverage and on the occurrence of movements beyond the home range, which was necessary to model the effects of weather on medium‐ and long‐range movements. Before estimating home ranges, we screened the data and removed implausible GPS locations falling more than 5 km away from the coast.

A complete overview of individuals from our sample is available in Table 1, while an extensive explanation about the release method and acclimatization is available in Fozzi et al. (2023).

**TABLE 1:** Overview of the total number of GPS locations, as well as the total number of GPS locations in medium‐range (MRMs) and long‐range movements (LRMs), for all 20 Griffon Vultures considered in our study. Further information on the same individuals is available in Fozzi et al. (2023) and Cerri et al. (2024).

Individual	GPS locations	Sex	GPS model	Date of birth	Date of release	Cohort	MRMs locations	LRMs locations
Artis 1	5485	M	Ecotone Crex	April 2017	2018‐04‐14	1	291	56
Artis 2	2810	F	Ecotone Crex	April 2017	2018‐04‐14	1	153	43
Artis 3	19,278	F	Ornitela 3G_50G	April 2018	2019‐06‐24	2	666	64
Artis 4	9309	M	Ornitela 3G_50G	April 2018	2019‐06‐24	2	156	227
Artis 5	2384	M	Ecotone Crex	April 2018	2019‐06‐24	2	91	44
Barca	15,784	F	Ecotone Skua	April 2015	2018‐04‐14	1	1227	358
Bulga	4618	F	Ecotone Crex	April 2015	2018‐04‐14	1	164	74
Calmedia	2816	F	Ecotone Crex	April 2018	2019‐10‐17	3	175	84
Caniga	8960	F	Ecotone Crex	April 2018	2019‐10‐17	3	290	214
Corte	4890	M	Ecotone Crex	April 2018	2019‐10‐17	3	209	135
Cristallo	13,505	M	Ecotone Crex	April 2015	2018‐04‐14	1	487	426
Doglia	18,090	F	Ornitela 3G_50G	April 2015	2019‐10‐17	3	468	274
Fenuggiu	10,255	M	Ecotone Crex	April 2015	2018‐04‐14	1	486	94
Macomer	8012	F	Ecotone Saker	April 2018	2019‐06‐24	2	488	72
Meilogu	2484	M	Ecotone Crex	April 2018	2019‐10‐17	3	103	3
Pabelanasa	10,526	F	Ecotone cDuck	April 2016	2018‐12‐12	4	614	251
Pituabile	6231	M	Ecotone Crex	April 2016	2018‐12‐12	4	222	73
Pozzomaggiore	6574	M	Ecotone Crex	April 2018	2019‐06‐24	2	680	108
Timidone	8451	M	Ecotone Crex	April 2018	2018‐04‐14	1	279	121
Tottubella	7740	F	Ecotone Crex	April 2018	2019‐10‐17	3	159	303

### 2.3 Classification of Movement Types

**FIGURE 2:**
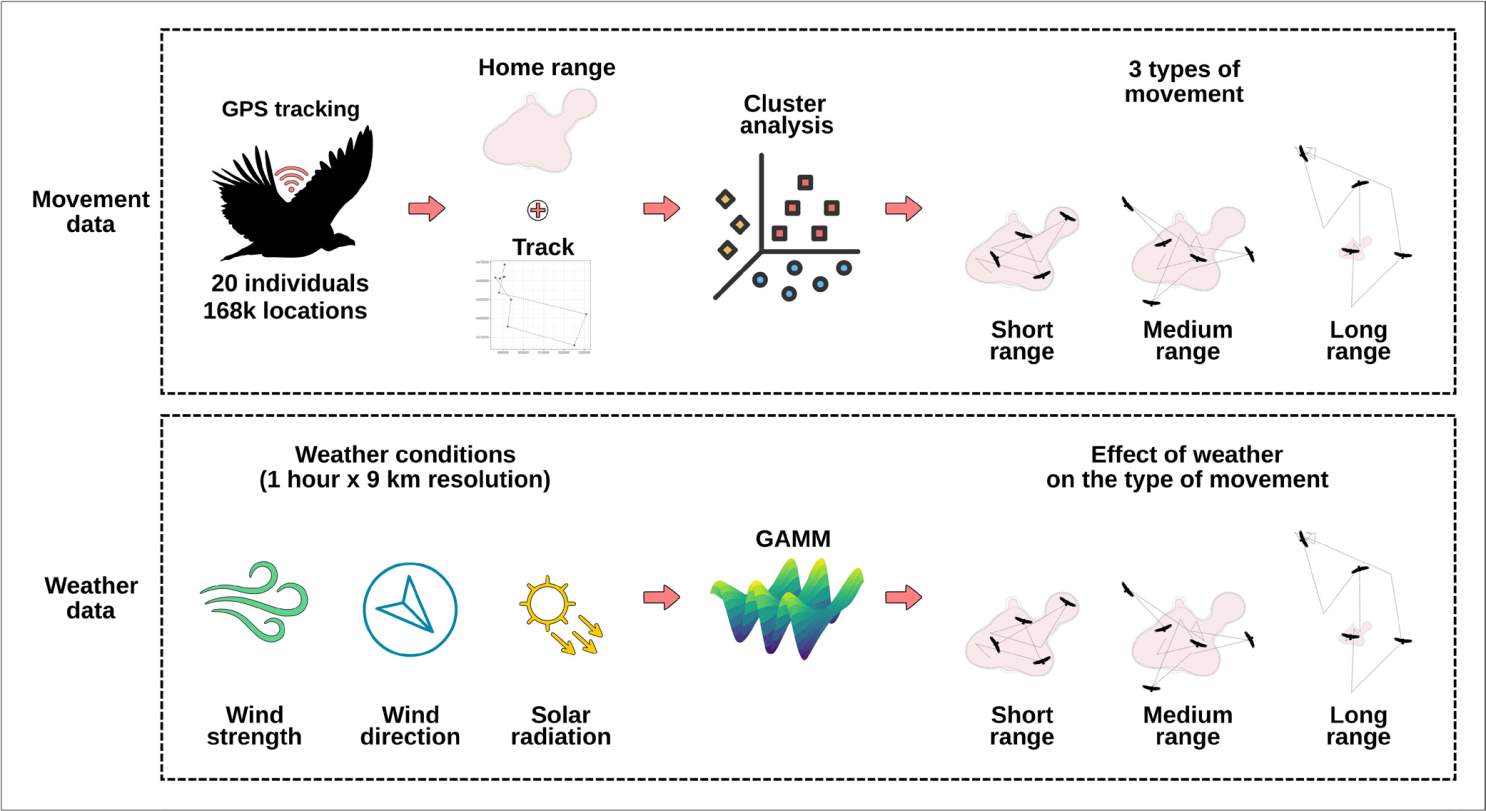
Workflow of data processing: Identification of the three types of movements from GPS telemetry data (top square) and integration of weather data to predict different types of movements (bottom square).

Movements were classified into short‐range movements (SRM), occurring within the home range, and movements occurring outside the home range, which were further classified as medium‐range (MRM) or long‐range movements (LRM) based on distance and duration (Figure 2). It is important to emphasize that, as Griffon Vultures in our study area do not have routine long‐range movements (e.g., due to migration or transhumance), both MRMs and LRMs identified nonroutine movements, whose ecological meaning will be discussed later in the manuscript.

The home range of each individual was quantified from the 95% isopleth of its range distribution (Alston et al. 2022), obtained through Autocorrelated Kernel Density Estimation (AKDE, Fleming et al. 2015; Fleming and Calabrese 2017) using continuous‐time movement models with a perturbative Hybrid REML (pHREML) estimator (Silva et al. 2022). We generally preferred weighted AKDE (wAKDE Silva et al. 2022) due to the irregular acquisition of GPS locations by solar‐powered tags and the lack of GPS location at night. Only for two individuals (Artis 4 and Artis 5), we used AKDE as wAKDE did not converge. Whenever a Griffon Vulture exited its home range, we isolated its movement track until the moment when the individual re‐entered its home range. Then, for each track movements outside the 95% home range we calculated: (i) the median step length, (ii) the median direction, expressed as degrees from the North, (iii) the maximum distance, expressed as the Euclidean distance between the most distant point of the track and the home range, (iv) its temporal duration, in hours and (v) the straightness index, expressed as the ratio between maximum distance and the total length of the track (Benhamou 2004). Step length and the straightness index were useful to distinguish between more directional and more diffusive movements (e.g., similarly to first‐passage‐time, Fauchald and Tveraa 2003), whereas the maximum distance and temporal duration of each track were useful to distinguish between forays and longer movements (Conradt et al. 2003). Finally, the direction of each track was used to potentially distinguish between long‐range movements occurring on different flyways and involving different parts of Sardinia. After having identified extra‐home range tracks and calculated the five‐movement metrics, we used Partitioning Around Medoids cluster analysis (PAM, Kassambara 2017) to divide them into different types of extra‐home range movements. Namely, PAM cluster analysis identified 2 different types of extra‐home range movements (Figure S2). These ultimately corresponded to medium‐range movements, where Griffon Vultures briefly exited their home range and then came back after a short period of time (Figure 1) and long‐range movements. LRMs had a significantly longer duration, higher movement tortuosity, and very high distances from the borders of the home range than medium‐range movements (Figure S3). At the end of this process, we therefore classified GPS locations as short‐range (inside the home range), medium‐range, and long‐range. The classification of extra‐home range trajectories by means of PAM clustering was favored over other methods for behavioral classification, due the irregular collection of GPS locations in conditions of low solar radiation.

Finally, for each GPS location, we extracted hourly values of solar radiation, as well as wind direction and strength, at a 10 km scale, from the ERA5 climate reanalysis dataset (https://cds.climate.copernicus.eu/cdsapp#!/dataset/reanalysis‐era5‐single‐levels). Solar radiation was quantified as the mean surface downward short‐wave radiation flux, representing the amount of solar radiation, both direct and diffuse, that reaches the ground. Downward short‐wave radiation also accounts for partial reflection and absorption by clouds and aerosols and corresponds to what would be measured by a pyranometer on the ground. Wind direction and speed were calculated from the eastward and northward components (ms‐1) of wind, at a height of 100 m above the surface of the Earth. Wind direction was expressed as degrees north, whereas wind speed in ms‐1. We also did not use different altitudinal layers, as our GPS tags did not provide the height of a certain GPS location.

### 2.4 Statistical Analyses

We used Generalized Additive Mixed Modeling (GAMM) to predict the probability that GPS locations belonged to SRMs, MRM, or LRMs. We treated our three types of observations (SRM, MRM, LRM) as an ordered response, which was modeled through a linear predictor providing the expected value of a latent variable following a logistic distribution. Predictors included different weather variables and individual attributes that we deemed to potentially influence nonroutine movements by Griffon Vultures.

We included wind direction, as it can influence different types of movements of Griffon Vultures in the study area. In northwest Sardinia, colonies are on the west coast and foraging ground in inner areas (Fozzi et al. 2025). Therefore, we hypothesized that Griffon Vultures could be more active in case of western winds that would allow them to reach foraging grounds, but which can subsequently drift them away (Tavecchia and Cortés‐Avizanda 2021), increasing the likelihood of MRMs and LRMs. In turn, we also included wind strength, because Griffon Vultures might be reluctant to fly around colonies with strong winds, as they can limit their aerial maneuverability around roosts (Shepard et al. 2019) or displace them on the open sea (Bildstein et al. 2009). We also accounted for solar radiation, as it is crucial for the generation of updrafts (Scacco et al. 2019) and therefore facilitates soaring flight in vultures (Fluhr et al. 2021; Poessel, Brandt, Mendenhall, et al. 2018; Rivers et al. 2014), promoting MRMs and LRMs.

We controlled for the release group of each individual, which was found to influence movement cohesion (Cerri, De Rosa, Aresu, et al. 2024; Cerri, De Rosa, Fozzi, et al. 2024) and therefore was believed to potentially influence their capacity to move across the landscape (Sassi et al. 2024). We also controlled for the age of each individual: as vultures age, they cover longer distances due to increased flight experience (Efrat et al. 2023; Harel et al. 2016), or changes in their movements due to age‐specific behaviors such as prospecting or breeding (Acácio et al. 2023). In turn, this could favor MRMs and LRMs. Additionally, we controlled for the sex of Griffon Vultures, as this variable was found to influence movement in individuals from populations of mainland Europe (Morant et al. 2023), and for the day of the year to capture long‐range movements caused by unmeasured seasonal variations in the environment (e.g., food, Arrondo et al. 2023; Spiegel et al. 2013). Finally, we added a random intercept for each individual to account for inter‐individual differences in the probability of MRMs and LRMs due to unobserved attributes (e.g., personality, Nilsson et al. 2014).

We modeled wind direction, the effect of the day of the year, and the effect of elapsed time since release by means of cyclic cubic splines, which are highly effective at capturing periodic patterns (Iannarilli et al. 2025). We rather used thin‐plate splines to model the effect of solar radiation and wind strength. We used a tensor product to model the interactive effect of wind strength and direction (Wood 2017). A complete script explaining model selection in GAMMs is provided in the Supporting Information.

We used a combination of likelihood ratio test, AIC, and generalized cross‐validation to select predictors and the numbers of basis in each spline. We selected the lowest number of bases after which we did not detect any improvement in model fitness to the data. Statistical analyses were carried out with the statistical software R (R Core Team 2025). Namely, GAMMs were fitted with the *mgcv* package (Wood 2017), and home ranges estimated with the *ctmm* package (Calabrese et al. 2016).

## 3 Results

Although most of our GPS locations belonged to SRMs (*n* = 157,770; 93.8%), we identified 4007 movement tracks and 7408 GPS locations associated with MRMs, as well as 593 movement tracks and 3024 GPS locations associated with LRMs.

Although our final candidate model had a low overall accuracy at predicting these three movements (Table 2), model selection clearly identified a set of covariates which were systematically associated with them and which progressively improved the goodness‐of‐fit of a certain model to the data (Table 2).

**TABLE 2:** Confusion matrix comparing observations classified as short‐range (SRM), medium‐range (MRM), and long‐range movements (LRM), with predicted values from the GAMM.

Behavior	SRM (predicted)	MRM (predicted)	LRM (predicted)
SRM (observed)	157,754	16	0
MRM (observed)	7397	11	0
LRM (observed)	3021	3	0

**FIGURE 3:**
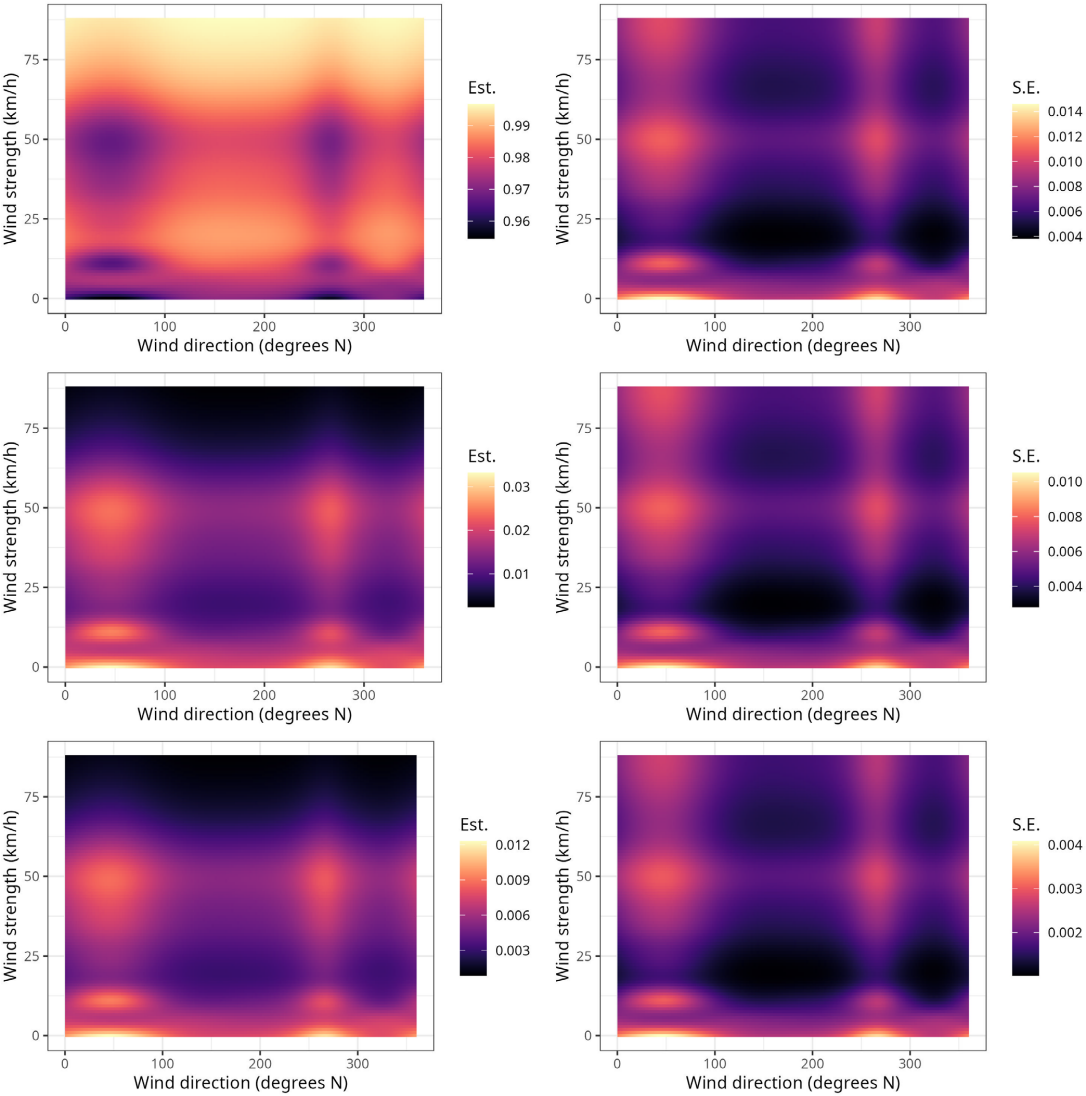
Estimated probabilities that GPS fix belonged to short‐range (top row), medium‐range (middle row), and long‐range movements (bottom row), according to wind strength and wind direction. Predicted probabilities are in the left column and standard errors in the right column. In the left column, lighter areas of the plot represent the conditions of wind strength and direction under which the probability that GPS fix belonged to a certain type of movement was the highest.

Griffon Vultures in our study area were more prone to engage in MRMs and LRMs when wind came from northwest (Mistral) and southeast (Sirocco). However, the effect of wind direction varied considerably according to wind strength (Figure 3). The probability of engaging in MRMs and LRMs for northwest and southeast wind showed three peaks: one for calm winds, (e.g., 0–2 km/h, according to the Beaufort scale, https://www.rmets.org/metmatters/beaufort‐wind‐scale), another one where for light breeze (e.g., 10–12 km/h) and a third one for strong breeze (e.g., 40–50 km/h).

Under extremely strong wind conditions (e.g., > 50 km/h), the effect of wind direction diminished, and Griffon Vultures only engaged in SRMs (Figure 3). SRMs were also generally common, regardless of wind direction, for gentle breeze (e.g., 15–20 km/h).

**FIGURE 4:**
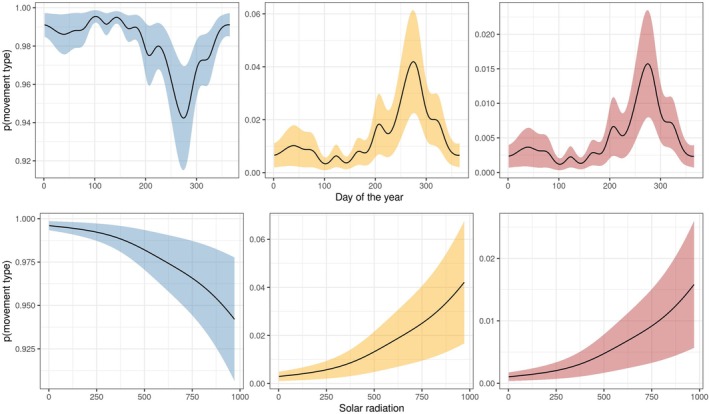
Marginal effect plot, showing the effect of the day of the year and solar radiation on the probability that GPS locations belonged to short‐range (left), medium‐range (middle), and long‐range movements (right).

The probability that Griffon Vultures engaged in MRMs and LRMs also increased markedly with higher solar radiation and in late summer (Figure 4). The age of released individuals also influenced this probability, with female Griffon Vultures increasing MRMs and LRMs after 5 years of age (Figure S4).

## 4 Discussion

To the best of our knowledge, this is the first study assessing weather conditions as a proximate driver of long‐range nonroutine movements in Old‐World vultures. Our findings align with those from studies finding that weather conditions affect the movements of large soaring raptors (*Aquila adalbertii*, Ferrer 1993; e.g., *A. chrysaetos* , Chaubet et al. 2025; Poessel et al. 2022; *Buteo buteo* , Walls et al. 2005; *Vultur gryphus* , Poessel, Brandt, Miller, and Katzner 2018 and Rivers et al. 2014). For example, we found that Griffon Vultures engage in longer movements when solar radiation is stronger and facilitates soaring flight (Scacco et al. 2019, 2023). However, our findings also highlight a nuanced interplay between solar radiation, wind direction, and wind strength: Griffon Vultures shift between different types of movement in response to specific conditions of wind and solar radiation.

SRMs, where Griffon Vultures move around the colony and foraging grounds (Cerri et al. 2023; Fozzi et al. 2025), happen under two particular weather conditions. The first one is whenever the wind has a speed of 15–20 km/h. Under these conditions, Griffon vultures probably manage to combine soaring flight and social cues to navigate the landscape around colonies and optimize their movements with respect to wind direction (Alerstam et al. 2019). However, Griffon Vultures also curtail their movements when wind speed exceeds 50 km/h, similarly to other large soaring birds facing dangerously strong winds (Naveda‐Rodríguez and Rush 2023; Wilkinson et al. 2019). We believe that future studies combining high‐resolution telemetry with accelerometers (Vaadia et al. 2025) will ultimately provide more accurate insights about SRMs and the optimization of flight strategies with respect to wind direction and strength (e.g., foraging, Hernandez‐Pliego et al. 2014, 2017; Cecere et al. 2020), also by linking movement patterns and wind conditions to foraging efficiency.

MRMs and LRMs are instead more common for northwestern and southeastern calm winds (0–2 km/h), light breezes (10–12 km/h), and strong breezes (e.g., 40–50 km/h). These three wind conditions probably facilitate a mixture of MRMs. Under calm winds or light breezes, Griffon vultures commute between the two colonies (Figure 1), forage westward of the main colony of Bosa (Fozzi et al. 2025) and engage in forays to discover the spatial distribution of resources (Conradt et al. 2003). Conversely, under strong breezes MRMs probably happen because Griffon Vultures are displaced from their home range by a few kilometers.

However, it is particularly interesting that LRMs begin under the same wind conditions as MRMs. Usually, Griffon Vultures would like to limit the duration of MRMs and return to the colony, but sometimes they fail to do so because wind is too strong and weather conditions change, limiting their motion capacity by reducing solar radiation (Scacco et al. 2019, 2023). At that point, they would decide to “go by the wind”, being thus further displaced and shifting from MRMs to LRMs, while attempting to re‐orientate and return. This would align with evidence showing that birds evaluate the practical feasibility of compensating for wind drift (e.g., during migration, Horton et al. 2016).

**FIGURE 5:**
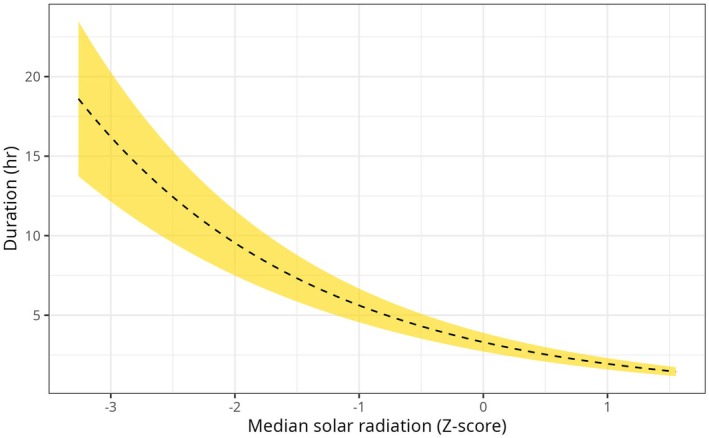
Marginal plot, for the GLMM showing the effect of median solar radiation experienced during a long‐range movement over its duration.

We tested for this potential explanation by fitting a Generalized Linear Mixed Model (GLMM) with a Gamma distribution to predict the duration of LRMs from solar radiation. Stepwise forward model selection was based on likelihood‐ratio testing (Table S1), and the best candidate model, although showing some patterns in its residuals (Figure S5), contained a random intercept accounting for variation between different individuals (Figure S6), while also accounting for the median solar radiation during LRMs and the age and release group of each individual (Figures S6, S7). In the best candidate model, the duration of LRMs has a strong negative association with solar radiation, with prolonged LRMs occurring when radiation was minimal (Figure 5). The effect of solar radiation did not differ between individuals of different age classes (Figure S8).

To the best of our knowledge, no study suggested displacement by wind as a mechanism driving long‐range nonroutine movements in large soaring birds, although it has been proven for several bird species during migration (Newton 2008) or foraging (e.g., seabirds, Hass et al. 2012; Weimerskirch and Prudor 2019). Our findings should encourage new studies about this topic, which can also address some limitations of our case study. For example, it would be important to replicate our findings in areas of mainland Europe, where colonies are more scattered across the landscapes, and Griffon Vultures can move isotropically across larger areas (Delgado‐González et al. 2022; Morant et al. 2023), unconstrained by the sea. In mainland populations, long‐range movements could also differ from those of our individuals, as colonies are often larger and Griffon Vultures can benefit from a higher number of social cues while navigating the landscape (Sassi et al. 2024). For gregarious vultures, such as the Griffon Vulture (van Overveld et al. 2020), future studies should also explore the joint spatial behavior of multiple individuals (Kaur et al. 2024) to test for potential differences between individual and collective nonroutine movements.

Moreover, although most of our tracked individuals were sexually immature at the time of the study, our results suggest that female Griffon Vultures increased LRMs after 5 years of age. Future studies, based on large samples of tracked Griffon Vultures tracked across their whole lifetime (Acácio et al. 2023), should therefore explore long‐term changes in the nonroutine movements due to age‐specific processes, such as sexual maturation and reproduction.

As the availability of high‐resolution weather data is increasing rapidly, we also believe that in a few years it will be possible to integrate them with high‐resolution telemetry (Carrard et al. 2025) and predict nonroutine long‐range movements in advance. By anticipating when vultures will engage in these movements, researchers can increase the acquisition rate of GPS tags to better understand habitat selection or energetic costs during important processes, such as forays (Poli et al. 2024), prospecting (Ponchon 2013), or dispersal (Orgeret et al. 2023).

## 5 Conclusions

Our findings indicate that the decision‐making of Griffon Vultures, while engaging in short and long‐range movements across the landscape, accounts for wind direction and strength, as well as for solar radiation. However, the most extreme long‐range movements occur when wind displaces Griffon Vultures and scarce solar radiation prevents them from returning to the colonies. At that point, individuals might engage in long journeys across unfamiliar landscapes while attempting to reorient. This indicates that some types of nonroutine movements are not entirely intentional, and weather conditions can play a crucial role in triggering them. In the future, combining high‐resolution movement and weather data could allow researchers to predict short and long‐range movements in advance and improve data acquisition from GPS tags adaptively to study vulture behavior during nonroutine movements and improve conservation actions.

## Author Contributions

**Jacopo Cerri:** formal analysis (lead), investigation (lead), methodology (lead), software (lead), supervision (lead), validation (lead), visualization (lead), writing – original draft (lead), writing – review and editing (lead). **Ilaria Fozzi:** data curation (equal), investigation (equal), validation (equal), writing – review and editing (equal). **Davide De Rosa:** data curation (lead), investigation (equal), validation (equal), writing – review and editing (equal). **Chiara Costantino:** formal analysis (supporting), methodology (supporting), validation (equal), writing – review and editing (equal). **Mauro Aresu:** data curation (equal), writing – review and editing (equal). **Dionigi Secci:** project administration (equal), resources (equal), writing – review and editing (equal). **Marco Muzzeddu:** funding acquisition (equal), resources (equal), writing – review and editing (equal). **Fiammetta Berlinguer:** funding acquisition (equal), investigation (equal), project administration (lead), resources (equal), supervision (equal), writing – review and editing (equal).

## Funding

This work was supported by LIFE Safe for Vultures, LIFE19 NAT/IT/000732; Ministero dell'Università e della Ricerca, DOT1629893‐2, UA2003DOTTRIC39_118.

## Disclosure

Declaration of Inclusion: The authors are committed to promoting diversity, equity, and inclusion in scientific research. In preparing this work, careful attention was given to the use of respectful and non‐discriminatory language. No contribution was excluded or diminished on the basis of age, gender, geographical origin, personal or professional condition, gender identity, belief, or ability. All co‐authors were granted equal opportunities to contribute to the ideas and content presented.

## Conflicts of Interest

The authors declare no conflicts of interest.

## Supporting information


**Appendix S1:** Overview of model selection for predicting the duration of LRMs.
**Figure S1:** Distribution of the time elapsed between consecutive GPS locations. Most GPS locations were collected every 50–70 min (left peak), but some every 110–125 min (right peak) due to irregularities connected with solar radiation and battery life.
**Figure S2:** Overview of the average silhouette method, the elbow method, and the gap statistics method to identify the optimal number of clusters for PAM cluster analysis. A complete description of the three methods is available in Kassambara (2017).
**Figure S3:** Comparison between the four groups of extra‐home range movement trajectories, identified by PAM cluster analysis. The four groups are compared in terms of their duration, the tortuosity, the maximum distance, the median step length, and their mean step direction.
**Figure S4:** Marginal effect plot, showing the effect of the age of each individual over the probability that male and female Griffon Vultures engaged in short‐range (left), medium‐range (middle), and long‐range movements (right).
**Figure S5:** Residuals of the best candidate model (mod.d) versus the median solar radiation (left), the age of each individual (center), and versus predicted values (right). The median solar radiation and the age of each individual are expressed as Z‐scores.
**Figure S6:** Estimated duration of long‐range movements (LRMs), according to the best candidate model (mod.d), between different groups of released Griffon Vultures and between different individuals.
**Figure S7:** Estimated duration of long‐range movements (LRMs), according to the best candidate model (mod.d), based on the age of released Griffon Vultures. The age is expressed as a Z‐score.
**Figure S8:** Estimated duration of long‐range movements (LRMs) according to the median solar radiation (as a Z‐score), between Griffon Vultures of different ages. The interaction term is from “mod.e” (see Table S1) and it was not significant according to likelihood ratio test, due to its low effect size.
**Table S1:** Overview of stepwise forward model selection, representing the Akaike's Information Criterion (AIC) and Un‐biased Risk Estimator (UBRE) of each model, as well as the chi‐squared and *p*‐values of likelihood‐ratio tests. Models were fitted with the “mgcv” package in R, by using a Gamma distribution of the response and Restricted Maximum Likelihood Estimation.

## Data Availability

Reproducible data and software code are available on GitHub at: https://github.com/JacopoCerri7/Nonroutine‐long‐range‐movements‐in‐Griffon‐Vultures and on Open Science Framework at: https://osf.io/qb5jr/.
